# Flagella by numbers: comparative genomic analysis of the supernumerary flagellar systems among the Enterobacterales

**DOI:** 10.1186/s12864-020-07085-w

**Published:** 2020-09-29

**Authors:** Pieter De Maayer, Talia Pillay, Teresa A. Coutinho

**Affiliations:** 1 School of Molecular & Cell Biology, University of the Witwatersrand, Wits, 2050 South Africa; 2 Centre for Microbial Ecology and Genomics, University of Pretoria, Pretoria, 0002 South Africa

**Keywords:** Enterobacterales, Supernumerary flagella, *Flag*, Evolution, Motility

## Abstract

**Background:**

Flagellar motility is an efficient means of movement that allows bacteria to successfully colonize and compete with other microorganisms within their respective environments. The production and functioning of flagella is highly energy intensive and therefore flagellar motility is a tightly regulated process. Despite this, some bacteria have been observed to possess multiple flagellar systems which allow distinct forms of motility.

**Results:**

Comparative genomic analyses showed that, in addition to the previously identified primary peritrichous (*flag*-1) and secondary, lateral (*flag*-2) flagellar loci, three novel types of flagellar loci, varying in both gene content and gene order, are encoded on the genomes of members of the order Enterobacterales*.* The *flag-*3 and *flag-*4 loci encode predicted peritrichous flagellar systems while the flag-5 locus encodes a polar flagellum. In total, 798/4028 (~ 20%) of the studied taxa incorporate dual flagellar systems, while nineteen taxa incorporate three distinct flagellar loci. Phylogenetic analyses indicate the complex evolutionary histories of the flagellar systems among the Enterobacterales.

**Conclusions:**

Supernumerary flagellar loci are relatively common features across a broad taxonomic spectrum in the order Enterobacterales. Here, we report the occurrence of five (*flag*-1 to *flag*-5) flagellar loci on the genomes of enterobacterial taxa, as well as the occurrence of three flagellar systems in select members of the Enterobacterales. Considering the energetic burden of maintaining and operating multiple flagellar systems, they are likely to play a role in the ecological success of members of this family and we postulate on their potential biological functions.

## Background

Flagella are complex structures that provide bacteria with an effective means of carrying out swimming (movement of single bacterial cells in liquid environments) and swarming (coordinated mobility of bacterial population on semi-solid or solid surfaces) movements [1]. Furthermore, they participate in biofilm formation, adhesion to surfaces and host cell invasion [2–4]. As such, flagella provide bacteria with a considerable competitive advantage over non-motile microorganisms occupying the same ecological niche and > 80% of known bacterial species are known to produce and maintain these structures [2, 3].

The basic structure of the flagellum is relatively well conserved among the flagellate bacteria, comprising of a basal body, hook and flagellar filament [5]. However, extensive diversity occurs within and between bacterial lineages in terms of the number of flagella per cell, the positions of the flagella on the cell surface, the number of genes required for the production and regulation of flagella [5, 6]. Bacterial flagellar arrangements vary from one polar flagellum (monotrichous), two polar flagella one at each end of the bacterial cell (amphitrichous), many polar flagella at one or both ends of the bacterial cell (lophotrichous), or several flagella distributed along the bacterial cell surface (peritrichous) [5]. In addition, a limited number of bacterial taxa produce dual flagellar systems encoded by distinct sets of genes present on the genome. This trait has been most widely studied in *Vibrio* and *Aeromonas* spp., which possess both a polar flagellum as well as several lateral (peritrichous) flagella [2, 6–9]. Dual flagellar systems have furthermore been observed in *Azospirillum, Rhodospirillum* and *Helicobacter* spp. [2]. The polar and lateral flagella allow these bacterial cells to carry out different modes of motility, where the polar flagellum facilitates swimming motility and the lateral flagella enable swarming motility [2, 6–9]. While this dual flagellar motility may provide the bacteria with a competitive advantage, flagellar synthesis and functioning is an energetically expensive commitment [3, 9]. As such, bacteria that are capable of producing dual flagellar systems tightly regulate the production of polar and lateral flagella [9, 10].

Members of the order Enterobacterales are commonly isolated from a wide range of environments including air, soil and water and include some of the most well-known pathogens of both plant and animal hosts [11, 12]. The ecological success of the Enterobacterales can in part be attributed to their capacity for motility, which is facilitated by their primary, peritrichous flagellar system [12, 13]. In the enterobacterial model organisms *Escherichia coli* and *Salmonella enterica*, up to 50 genes are required the assembly, maintenance and functioning of this flagellum. These genes are organized in three genomic clusters, which are collectively termed the primary flagellar (*flag*-1) locus [3, 12, 13]. In addition to the *flag*-1 system, a second, evolutionary distinct, flagellar system, encoded by the *flag*-2 locus, was observed and shown to be relatively prevalent among members of the order Enterobacterales [14, 15]. The *flag*-2 locus encodes a lateral flagellar system and has been postulated to facilitate swarming motility among the Enterobacterales, although it has been inactivated through gene deletions and transposon integration in a substantial proportion of enterobacterial taxa [14–16]. Here, by means of a comprehensive comparative genomic analysis, we identified three additional distinct flagellar loci *flag*-3, *flag*-4 and *flag*-5 which are distributed among the Enterobacterales. These occur predominantly as a secondary flagellar system in enterobacterial taxa with the *flag*-1 primary flagellar system, while a limited subset of taxa incorporate three flagellar loci. Here we postulate on the evolutionary histories and potential functions of these supernumerary flagellar systems among the Enterobacterales.

## Results

### The Enterobacterales encode five distinct flagellar systems

**Fig. 1:**
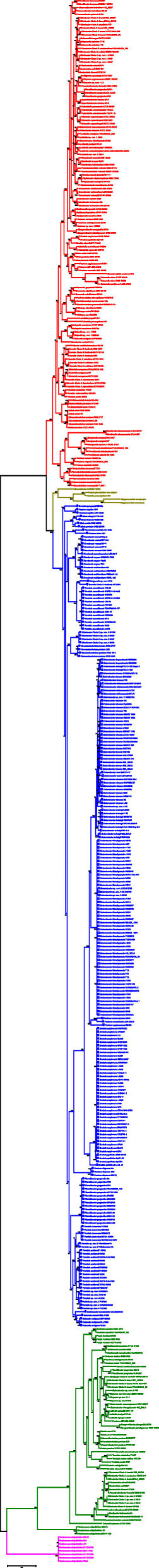
Phylogenetic differentiation of five distinct flagellar (*flag*) loci among the Enterobacterales. A ML phylogeny was constructed on the basis of 25 SCOs conserved among all *flag* loci. The trimmed alignment was comprised of 4523 amino acid sites and the best-fit evolutionary model LG + F + I + G4 was selected for phylogeny construction. Bootstrap values (*n* = 1000) are shown. The *flag*-1 (red), *flag*-2 (green), *flag*-3 (blue), *flag*-4 (olive) and *flag*-5 (pink) clades are indicated by the distinct branch colours

**Fig. 2:**
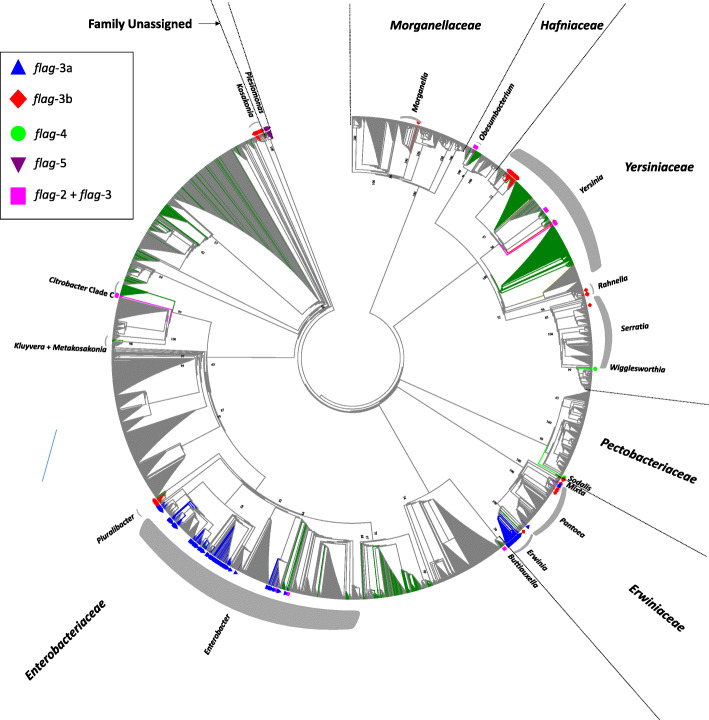
Taxonomic distribution of the novel *flag*-3, *flag*-4 and *flag*-5 loci among the Enterobacterales. The phylogeny was constructed as previously described [15] on the basis of four house-keeping proteins, GyrB, InfB, RecA and RpoB. The trimmed concatenated alignment comprised of 2613 amino acid sites and the tree was constructed using the best-fit evolutionary model JTTDCMut+I + G4. Bootstrap support (n = 1000 replicates) values > 50% are indicated for the major clades. The presence of the different loci are indicated by different coloured shapes, blue triangles (*flag*-3a), red diamonds (*flag*-3b), green circles (*flag*-4) and purple triangles (*flag*-5), while those taxa where both *flag*-2 and *flag*-3 loci are present are indicated by pink squares. While the prevalence of *flag*-1 loci is not shown, those taxa that incorporate *flag*-2 loci are indicated by green-coloured branches

The complete and draft genomes of 4082 taxa belonging to the order Enterobacterales were screened for the presence of additional flagellar (*flag*) loci by performing tBlastN analysis of the full complement of complete protein sequences required for the synthesis of the *flag*-1 and *flag*-2 flagella of *E. coli* K-12 strain MG1655 (47 proteins) and *E. coli* 042 (38 proteins), respectively, against the genome sequences. In total, 816 (20.26%) of the studied strains incorporate at least two distinct *flag* loci (Table 1; Additional file 1: Table S1). When considering that 664 strains lack any *flag* loci altogether and can be considered as incapable of swimming motility, dual or multiple *flag* loci are hallmarks of 24.26% of the presumed motile enterobacterial taxa. The secondary *flag* loci are predominated by the previously characterized *flag*-2 loci, with 593 (575 strains with and 18 strains lacking *flag*-1 loci) of the studied strains incorporating this locus (Table 1) [15]. The protein complements encoded by the remaining additional flagellar loci, along with taxonomically representative *flag*-1 and *flag*-2 datasets were compared. A total of twenty-five distinct single-copy orthologues (SCOs) are conserved among the loci and were aligned and concatenated to generate a Maximum Likelihood (ML) phylogeny. The resultant SCO phylogeny showed that, aside from the *flag*-1 and *flag*-2 loci, the remaining loci form three distinct clades, which we have termed *flag-*3, *flag*-4 and *flag*-5 loci (Fig. 1). The largest clade incorporates the novel *flag*-3 loci, which occur in 249 distinct taxa (6.18% of total taxa studied), across a broad taxonomic spectrum, including members of the families *Enterobacteriaceae Erwiniaceae, Hafniaceae*, *Morganellaceae* and *Yersiniaceae* (Table 1; Fig. 2). By contrast, the *flag*-4 and *flag*-5 loci are less prevalent, being restricted to six (four *Pectobacteriaceae* and two *Yersiniaceae* members) and eight taxa (all *Plesiomonas shigelloides* isolates; Family unassigned), respectively (Table 1; Fig. 2).

**Table 1:** Prevalence of the primary (*flag*-1) and additional (*flag*-2 to *flag*-5) loci among the Enterobacterales**.** The number and percentage of taxa at the family and genus level that incorporate each type of locus are indicated

**Family**	**Genus**	**# Strains**	***flag-*1**	***flag-*2**	***flag-*3a**	***flag-*3b**	***flag-*4**	***flag-*5**	***# Strains with*** **3** ***flag*** **loci**	
***Budviciaceae***		**9**	**0 (0%)**	**7 (77.8%)**	**0 (0%)**	**0 (0%)**	**0 (0%)**	**0 (0%)**		
	*Budvicia*	2	–	2 (100%)	–	–	–	–		
	*Leminorella*	2	–	1 (50%)	–	–	–	–		
	*Limnobaculum*	1	–	–	–	–	–	–		
	*Pragia*	4	–	4 (100%)	–	–	–	–		
***Enterobacteriaceae***		**2464**	**1878 (76.2%)**	**317 (12.9%)**	**110 (4.5%)**	**34 (1.4%)**	**0 (0%)**	**0 (0%)**	**7 (0.3%)**	
	*Atlantibacter*	5	5 (100%)	–	–	–	–	–		
	*Buttiauxella*	8	8 (100%)	4 (50%)	–	1 (12.5%)	–	–	1 (12.5%)	
	*Cedecea*	8	8 (100%)	6 (75%)	–	–	–	–		
	*Citrobacter* Clade A	157	157 (100%)	91 (58.0%)	–	–	–	–		
	*Citrobacter* Clade B	21	21 (100%)	5 (23.8%)	–	–	–	–		
	*Citrobacter* Clade C	4	4 (100%)	4 (100%)	–	4 (100%)	–	–	4 (100%)	
	*Citrobacter* Clade D	30	30 (100%)	30 (100%)	–	–	–	–		
	*Cronobacter*	189	189 (100%)	–	–	–	–	–		
	*Enterobacter*	608	608 (100%)	30 (4.9%)	110 (18.1%)	–	–	–	2 (0.3%)	
	*Escherichia*	522	522 (100%)	124 (23.8%)	–	–	–	–		
	*Franconibacter*	10	10 (100%)	–	–	–	–	–		
	*Klebsiella* Clade A	100	100 (100%)	–	–	–	–	–		
	*Klebsiella* Clade B	310	–	–	–	–	–	–		
	*Klebsiella* Clade C	189	–	–	–	–	–	–		
	*Kluyvera*	9	9 (100%)	4 (44.4%)	–	–	–	–		
	*Kosakonia*	24	24 (100%)	–	–	15 (62.5%)	–	–		
	*Leclercia*	10	10 (100%)	–	–	–	–	–		
	*Lelliottia*	13	13 (100%)	9 (69.2%)	–	–	–	–		
	*Mangrovibacter*	2	2 (100%)	2 (100%)	–	–	–	–		
	*Metakosakonia*	3	3 (100%)	1 (33.3%)	–	–	–	–		
	*Phytobacter*	2	2 (100%)	–	–	–	–	–		
	*Pluralibacter*	17	17 (100%)	2 (11.8%)	–	14 (82.4%)	–	–		
	*Pseudescherichia*	2	2 (100%)	2 (100%)	–	–	–	–		
	*Pseudocitrobacter*	1	1 (100%)	1 (100%)	–	–	–	–		
	*Raoultella*	85	–	–	–	–	–	–		
	*Salmonella*	112	112 (100%)	–	–	–	–	–		
	*Shimwellia*	2	–	–	–	–	–	–		
	*Siccibacter*	6	6 (100%)	2 (0.33%)	–	–	–	–		
	*Superficieibacter*	2	2 (100%)	–	–	–	–	–		
	*Trabulsiella*	8	8 (100%)	–	–	–	–	–		
	*Yokenella*	5	5 (100%)	–	–	–	–	–		
***Erwiniaceae***		**287**	**279 (97.2%)**	**8 (2.8%)**	**44 (15.3%)**	**12 (4.2%)**	**2 (0.7%)**	**0 (0%)**		
	*Buchnera*	58	58 (100%)	–	–	–	–	–		
	*Erwinia*	63	60 (95.2%)	2 (3.2%)	41 (65.1%)	1 (1.6%)	–	–		
	*Izhakiella*	2	2 (100%)	2 (100%)	–	–	–	–		
	*Mixta*	4	4 (100%)	–	–	4	–	–		
	*Pantoea*	151	151 (100%)	2 (1.3%)	3 (2%)	7 (4.6%)	–	–		
	*Phaseolibacter*	1	–	–	–	–	–	–		
	*Rosenbergiella*	1	1 (100%)	1 (100%)	–	–	–	–		
	*Tatumella*	5	3 (60%)	1 (20%)	–	–	–	–		
	*Wigglesworthia*	2	–	–	–	–	2	–		
***Hafniaceae***		97	**97 (100%)**	**28 (28.9%)**	**0 (0%)**	**4 (4.1%)**	**0 (0%)**	**0 (0%)**	**4 (4.1%)**	
	*Edwardsiella*	50	50 (100%)	–	–	–	–	–		
	*Enterobacillus*	1	1 (100%)	–	–	–	–	–		
	*Hafnia*	42	42 (100%)	24 (57.1%)	–	–	–	–		
	*Obesumbacterium*	4	4 (100%)	4 (100%)	–	4	–	–	4 (100%)	
***Morganellaceae***		**313**	**311 (99.4%)**	**0 (0%)**	**0 (0%)**	**1 (0.3%)**	**0 (0%)**	**0 (0%)**		
	*Arsenophonus*	2	2 (100%)	–	–	–	–	–		
	*Moellerella*	2	2 (100%)	–	–	–	–	–		
	*Morganella*	55	55 (100%)	–	–	1 (1.8%)	–	–		
	*Photorhabdus*	31	31 (100%)	–	–	–	–	–		
	*Proteus*	122	122 (100%)	–	–	–	–	–		
	*Providencia*	58	56 (96.6%)	–	–	–	–	–		
	*Xenorhabdus*	43	43 (100%)	–	–	–	–	–		
***Pectobacteriaceae***		244	**241 (98.8%)**	**0 (0%)**	**0 (0%)**	**0 (0%)**	**4 (1.6%)**	**0 (0%)**		
	*Biostraticola*	1	–	–	–	–	1 (100%)	–		
	*Brenneria*	9	9 (100%)	–	–	–	–	–		
	*Dickeya*	55	55 (100%)	–	–	–	–	–		
	*Lonsdalea*	35	35 (100%)	–	–	–	–	–		
	*Pectobacterium*	140	138 (98.6%)	–	–	–	–	–		
	*Samsonia*	1	1 (100%)	–	–	–	–	–		
	*Sodalis*	3	3 (100%)	–	–	–	3 (100%)	–		
***Thorselliaceae***		**1**	**1 (100%)**	**0 (0%)**	**0 (0%)**	**0 (0%)**	**0 (0%)**	**0 (0%)**		
	*Thorsellia*	1	1 (100%)	–	–	–	–	–		
***Yersiniaceae***		**605**	**593 (97.9%)**	**225 (37.2%)**	**0 (0%)**	**44 (7.3%)**	**0 (0%)**	**0 (0%)**	**8 (1.3%)**	
	*Chania*	2	2 (100%)	1 (50%)	–	–	–	–		
	*Ewingella*	2	2 (100%)	–	–	–	–	–		
	*Gibbsiella*	4	–	–	–	–	–	–		
	*Nissabacter*	1	1 (100%)	–	–	–	–	–		
	*Rahnella*	18	17 (94.4%)	–	–	4 (22.2%)	–	–		
	*Rouxiella*	3	–	1 (33.3%)	–	–	–	–		
	*Serratia* Clade A	159	157 (98.7%)	–	–	–	–	–		
	*Serratia* Clade B	12	12 (100%)	–	–	1 (8.3%)	–	–		
	*Serratia* Clade C	8	8 (100%)	–	–	–	–	–		
	*Serratia* Clade D	1	–	–	–	–	–	–		
	*Serratia* Clade E	1	–	–	–	–	–	–		
	*Yersinia*	394	394 (100%)	223 (56.6%)	–	39 (10%)	–	–	8 (2.0%)	
**Family Unassigned**		**8**	**0 (0%)**	**8 (100%)**	**0 (0%)**	**0 (0%)**	**0 (0%)**	**8 (100%)**		
	*Plesiomonas*	8	–	8 (100%)	–	–	–	8 (100%)		
**OVERALL**		**4028**		**3400 (84.4%)**	**593 (14.7%)**	**154 (3.8%)**	**95 (2.4%)**	**6 (0.1%)**	**8 (0.2%)**	**19 (0.5%)**

The *flag*-3 loci are predominantly found in enterobacteria that also harbour a *flag*-1 locus, with only *Rahnella variigena* DLL 7529 incorporating a *flag*-3 locus, but lacking the *flag*-1 system. This is similar to the *flag*-2 system which, with the exception of ten taxa, occur in *flag*-1 flagellated taxa [15]. The *flag*-4 system occurs in three taxa (*Sodalis* spp.) that also incorporate *flag*-1 loci, while the other three (two *Wigglesworthia* spp. and *Biostraticola tofi* DSM 19580) lack the latter locus. All *flag*-5 encoding *P. shigelloides* strains also incorporate a *flag*-2 locus, but lack a *flag*-1 locus. While the majority of enterobacteria with supernumerary flagellar loci incorporate two *flag* loci in their genome, nineteen taxa (~ 0.5% of all strains studied) incorporate three distinct *flag* loci (Table 1). Eighteen of these harbour a *flag*-1, *flag*-2 and *flag*-3 locus and include members of the families *Enterobacteriaceae* (one strain of *Buttiauxella warmboldiae*, one strain of *Enterobacter* and four strains of *Citrobacter* Clade C), *Hafniaceae* (four strains of *Obesumbacterium proteus*) and *Yersiniaceae* (eight *Yersinia* spp.) (Table 1; Fig. 2). One strain, *Enterobacter ludwigii* OLC-1682, lacks a *flag*-2 locus, but instead incorporates one *flag*-1 locus and two *flag*-3 loci (discussed in further detail below).

**Fig. 3:**
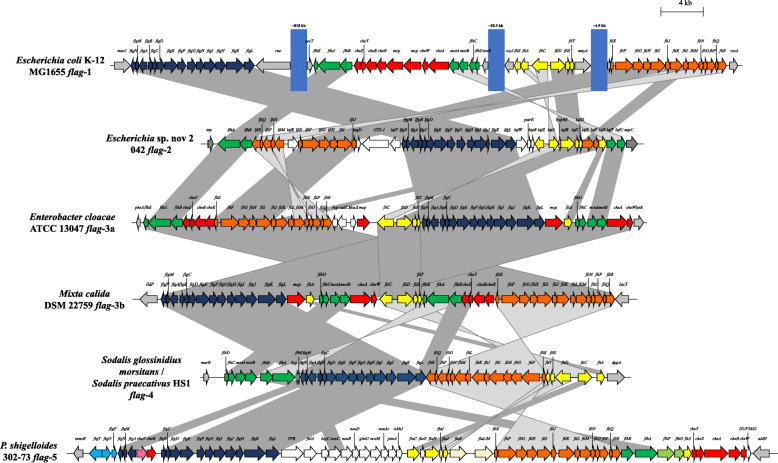
Schematic comparison of the *flag*-1 to *flag*-5 loci. The *flag* loci genes are coloured according to orthology to conserved genes in the *flag*-1 locus, with flanking genes coloured in grey, and non-conserved cargo genes in white. The blue blocks indicate chromosomal gaps between the *flag*-1 locus regions. The scale bar (4 kilobases) indicates the relative sizes of the loci

Comparison of the *flag*-1 through to *flag*-5 loci revealed that, while there is some synteny in gene blocks between the distinct *flag* loci (*flag*-1 through to *flag*-5), there is also evidence of extensive rearrangements and inversion of gene blocks (Fig. 3). Furthermore, some of the *flag* loci show evidence of gene deletion events, while in others additional, non-conserved genes have been integrated within the loci (Fig. 3). Average amino acid identity (AAI) values across the 25 SCOs shared among all *flag* loci further supports the separation of the enterobacterial *flag* loci into five distinct types with intra-clade AAI values ranging between 57.79% (*flag*-4 loci) and 99.06% (*flag*-5 loci), while the inter-clade values are only between 32.80 and 52.23% (Additional file 1: Table S2). These data suggest distinct evolutionary histories for the *flag*-1 to *flag-*5 loci. As such, a more in depth analysis of the *flag*-3, − 4 and − 5 loci was undertaken.

### The *flag*-3 peritrichous flagellar loci can be further divided into two subtypes, *flag*-3a and *flag*-3b on the basis of sequence synteny and conservation

The *flag*-3 loci cluster with the peritrichous primary flagellar (*flag*-1) loci in the SCO phylogeny (Fig. 1) and they share 52.23% average amino acid identity across the twenty-five conserved SCO proteins encoded on the loci. The former loci incorporate genes coding for 46/47 proteins involved in flagellar biosynthesis, regulation and maintenance and chemotaxis in the primary flagellar locus. One exception is the absence of orthologues of the gene coding for FliZ in the *flag*-3 loci. In the *flag*-1 loci FliZ is an activator for the expression of class 2/middle genes involved in the synthesis in the flagellar hook and basal body [17]. A distinct regulatory system for class 2 gene expression may occur in the *flag*-3 system.

**Fig. 4:**
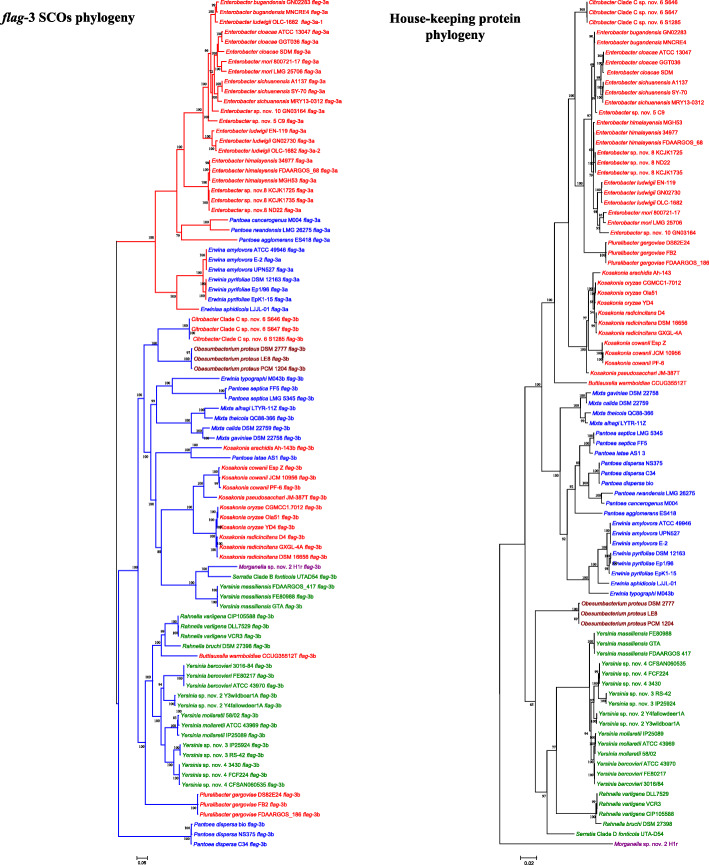
Phylogeny of *flag*-3 loci compared to house-keeping protein tree. ML phylogenies were constructed on the basis of the concatenated alignments of 45 SCOs conserved among the *flag*-3 loci (left) and the house-keeping proteins GyrB, InfB, RecA and RpoB (right). The final alignments comprised of 10,357 and 3086 amino acids, respectively. The best-fit evolutionary model LG + F + I + G4 was used for construction of both trees and bootstrap support (n = 1000 replicates) values are shown. The taxa names are coloured according to their family designation, while the *flag*-3a and *flag*-3b loci branches are coloured in red and blue, respectively in the *flag*-3 SCO phylogeny

Phylogenetic evaluation of the *flag*-3 loci, on the basis of the concatenated alignment of 45 SCOs (excluding FliC which is present in multiple copies in some strains) conserved amongst them, showed that they fall into two distinct clades (Fig. 4), with the loci of the upper clade (*flag*-3a) comprising 154 (61.6% of total *flag*-3 loci) and the lower clade (*flag*-3b) comprising 95 taxa. The *flag*-3a loci are restricted to members of three genera in two enterobacterial families (the *Enterobacteriaceae* and *Erwiniaceae*), namely *Enterobacter* (111/608 of the studied strains), *Erwinia* (41/61 of the studied taxa) and *Pantoea* (3/151 studied taxa) (Table 1). By contrast, the *flag*-3b locus is represented across a much broader taxonomic spectrum, including the *Enterobacteriaceae* (one *Buttiauxella,* four *Citrobacter* Clade D, fifteen *Kosakonia* and fourteen *Pluralibacter* strains), *Erwiniaceae* (one *Erwinia*, three *Mixta* and seven *Pantoea* strains), *Hafniaceae* (four *Obesumbacterium proteus* strains), *Morganellaceae* (*Morganella* sp. nov. 2 H1r) and *Yersiniaceae* (four *Rahnella*, one *Serratia* Clade B and 39 *Yersinia* strains) (Table 1). The presence of these *flag*-3 loci appears to be mutually exclusive, with no taxon containing both *flag*-3a and *flag*-3b loci. There is, however, one strain, *Enterobacter ludwigii* OLC-1682 which incorporates two *flag*-3a loci. These cluster together with the other *Enterobacter* sp. *flag*-3a loci, but in distinct sub-clades, with *flag*-3a-1 clustering with those of *E. bugandensis* and *flag*-3a-2 clustering with the other *E. ludwigii* loci. They furthermore share 84.42% AAI values (entire *Enterobacter* clade = 84.12% AAI), suggesting that *E. ludwigii* OLC-1682 derived these loci through distinct evolutionary events.

**Fig. 5:**
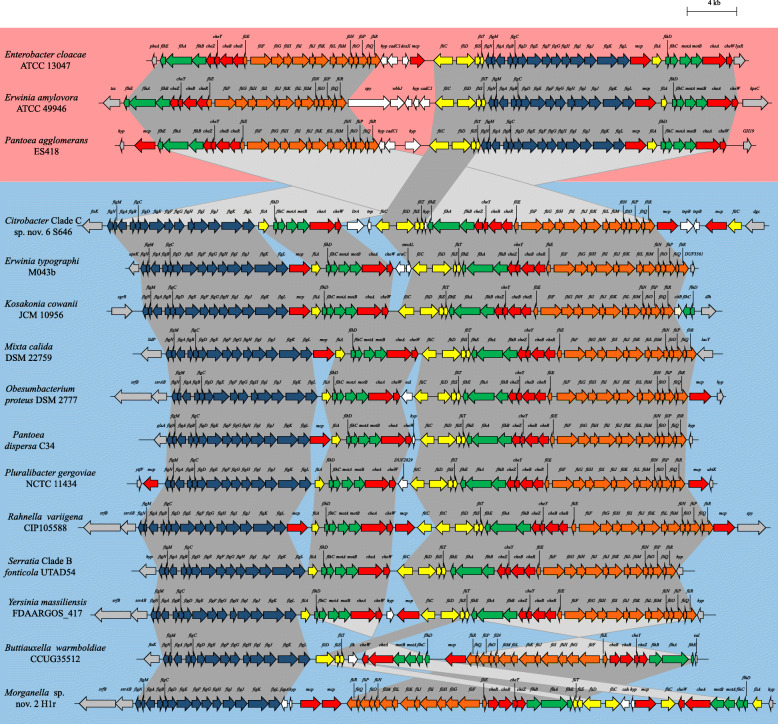
Schematic comparison of the *flag*-3 loci. The *flag* loci genes are coloured according to orthology to conserved genes in the *flag*-1 locus, with flanking genes coloured in grey, and non-conserved cargo genes in white. The *flag*-3a loci are background shaded pink, while the *flag*-3b loci are shaded light blue. The scale bar (4 kilobases) indicates the relative sizes of the loci

The *flag*-3a loci are on average 46.3 kb in size, have an average G + C content of 55.0% and code for 51 distinct proteins, while the *flag-*3b loci are ~ 44.3 kb in size, have an average G + C content of 50.5% and code for 48 proteins (Additional file 1: Table S3). The proteins encoded on the two subtypes, *flag*-3a and *flag*-3b, share distinct sequence conservation, with intraclade AAI values of 75.54% (*flag*-3a) and 69.09% (*flag*-3b), respectively, while the inter-clade AAI value is 59.01% (on the basis of 25 conserved SCOs) (Additional file 1: Table S2). Furthermore, the *flag*-3a and *flag*-3b loci have distinct gene syntenies. The *flag*-3a loci all comprise of three gene blocks occurring in the order block 1: *flhEAB-cheZYBR-fliEFGHIJKLMNOPQR*, block 2: *fliCDST* and block 3: *flgNMABCDEFGHIJKL-fliA-flhDC-motAB-cheAW*. In most of the *flag*-3b loci block 3 precedes block 2 and block 1 is situated at the 3′ end of the locus. Two notable exceptions are the *flag*-3b loci of *Buttiauxella warmboldiae* CCUG 35512 and *Morganella* sp. nov 2 H1r. In both taxa, gene block 3 occurs at the 5′ end of the locus, but block 2 is integrated within gene block 1, with part of the latter gene block occurring in reverse complement (Fig. 5). The co-localisation of the genes in their gene blocks, regardless of the locus subtype, suggests these loci may have been built by the step-wise incorporation of the individual gene blocks, which may have been derived from distinct ancestral loci. This is supported by the distinct G + C contents of the gene blocks. The G + C content of gene block 1 is on average 4.45 and 2.23% higher than those of block 2 and block 3 of the *flag*-3 loci (both *flag*-3a and *flag*-3b), respectively.

Similar to what is observed in both *flag*-1 and *flag*-2 loci [12, 15], the *flag*-3a and *flag*-3b loci are characterized by the presence of a non-conserved genomic island adjacent to the *fliC* gene (Fig. 5). This island occurs in 150/155 of the *flag*-3a loci and 78/95 of the *flag*-3b loci, has an average size of 3.9 kb (range: 0.5–8.9 kb) and codes for between one and eight distinct cargo proteins (Additional file 1: Table S3). A total of twenty-seven distinct proteins are encoded on this island, with nine of these unique to the *flag*-3a loci and seventeen unique to the *flag*-3b loci (Additional file 1: Table S4). One island feature is shared between the islands of 15 *flag*-3a and 57 *flag*-3b loci and codes for a methyl-accepting chemotaxis protein (Mcp1; 45.26% average amino acid identity; COG0840). A second methyl-accepting chemotaxis protein (Mcp2; 86.22% average amino acid identity; PRK15048) is found in the *flag*-3a islands of *Enterobacter* and *Pantoea* spp. A key feature among the Enterobacterales is the presence of genes in the flagellar locus coding for proteins with roles in glycosylation and post-translational modification of the main flagellar structural protein, flagellin. Flagellin glycosylation has been linked to a number of functions, including flagellar synthesis and stabilization, biofilm formation, surface recognition and adherence, virulence and host immune evasion [18, 19]. Previous studies that showed 17.4% (307/1761) and 57.6% (341/592) of the *flag*-1 and *flag*-2 loci incorporated flagellin glycosylation machinery, respectively [12, 15]. Among the *flag*-3 loci, only the *flag*-3a loci of 41 (26.45% of the *flag*-3a loci; 16.4% of total *flag*-3 loci) *Erwinia* spp. incorporate three genes coding for enzymes involved in flagellin modification adjacent to the *fliC* gene (Fig. 5). One gene codes for a 1127 amino acid (96.45% AAI) N-acetyl glucosamine glycosyltransferase (Spy), which catalyses the post-translational addition of *O-*linked beta-N-acetylglucosamine to serine/threonine residues in the target protein (Additional file 1: Table S4) [20]. The flagellin glycan chains are frequently further decorated by formyl, methyl, acetyl and amino groups [12]. Adjacent to the *spy* gene in the *Erwinia flag*-3a are genes coding for an aminotransferase (WecE; 98.01% AAI; COG0399) and *O-*acetyltransferase (WbbJ; 93.65% AAI; COG0110) suggesting that the *Erwinia flag*-3b flagellin glycan is both acetylated and aminated, while the Spy protein also incorporated a methyltransferase domain (pfam13649) (Additional file 1: Table S4).

A key feature of the non-conserved island adjacent to the *fliC* gene among the *flag*-3a loci is the universal presence of a gene coding for a transcriptional regulator, CadC1 (COG3710) (Additional file 1: Table S4). Given its position in the locus, where *fliZ* occurs in the *flag*-1 locus, it is plausible that this transcriptional regulator may serve a similar role in the *flag*-3a loci, but this will need to be validated experimentally. No gene with this purported function is present in the *flag*-3b loci and hence how the class 2 gene expression would be regulated in the latter loci remains unclear.

### The *flag*-4 locus is predominant among insect endosymbionts and codes for a predicted peritrichous flagellum

**Fig. 6:**
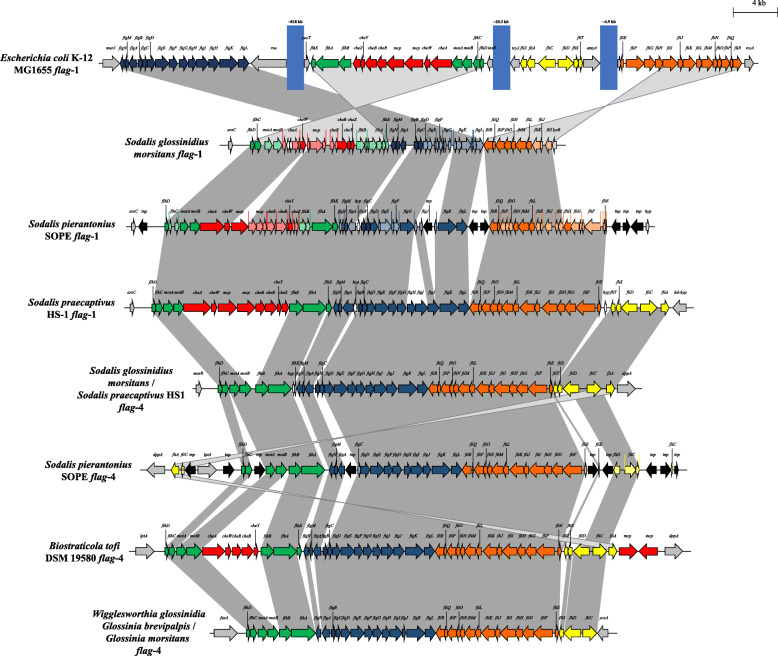
Schematic comparison of the *flag*-4 loci and *Sodalis flag*-1 loci. The *flag* loci genes are coloured according to orthology to conserved genes in the *flag*-1 locus, with flanking genes coloured in grey, non-conserved cargo genes in white and transposase genes in black. Predicted pseudogenes are shown as hashed arrows. The blue blocks indicate chromosomal gaps between the *flag*-1 locus regions. The scale bar (4 kilobases) indicates the relative sizes of the loci

The *flag*-4 loci cluster with the *flag*-1 and *flag*-3 loci in the flagellar SCO phylogeny and share 49.69 and 46.73% AAI values with the former and latter loci, respectively (Fig. 1; Additional file 1: Table S2). BlastP analysis of the *flag-*4 protein complement revealed the closest matches are proteins encoded on the *flag*-1 and *flag*-3 loci, suggesting that the *flag*-4 locus likewise codes for a peritrichous flagellar system. Of all the *flag* systems, the *flag*-4 loci show the greatest versatility, ranging in size between 31.6 and 45.1 kb and G + C content between 22.5 and 56.1% with an intra-clade AAI value of 57.79% (across 25 conserved SCOs) (Additional file 1: Tables S2 and S3). Furthermore, while the *flag*-4 loci share extensive synteny, the *flag-*4 loci show evidence of frequent deletions and gene disruption through transposon integration (Fig. 6). As such, the *flag*-4 locus of the tsetse fly endosymbiont *Sodalis glossinidius* ‘morsitans B4’ lacks the *cheZYBR-cheAW* and *fliZ* genes and incorporates a pseudogene of *flhE*, while that of *S. pierantonius* SOPE (*Sitophilus oryzae* endosymbiont) lacks the genes *cheZYBR-cheAW*, *flhE*, *flgB*, *fliZST* and incorporates pseudogene copies of *flgC*, *fliQ, fliE, fliC* and *fliD*. Similarly, while their genomes incorporate *flag-*1 loci, they are likewise heavily degraded (Fig. 6). The eroded *flag*-1 and *flag*-4 loci in these *Sodalis* strains are typical of the observed degenerative genome evolution as these bacteria adapted to a symbiotic lifestyle and indeed they have been described as non-motile [21, 22]. The gene complement of the *flag*-4 locus of *Sodalis praecaptivus* HS1 reflects that observed in *S. glossinidius* ‘morsitans B4’, and it also harbours a complete *flag-*1 locus (encodes 46/47 of the primary flagellar proteins, with the exception of FliZ). This latter strain, differs from the insect symbiont *Sodalis* spp. in that it was isolated from a human wound, can persist in free-living form and has been observed to be capable of swarming motility [23, 24], although whether the *flag*-1 or *flag*-4 locus encoded flagellar system is responsible for this capacity remains to be elucidated.

The two tsetse fly endosymbiotic *Wigglesworthia glossinidia* strains included in this study lack *flag*-1 loci but both incorporate *flag*-4 loci. The loci of these two strains are the smallest among the *flag*-4 loci (Additional file 1: Table S3) and include only 33/47 genes coding for orthologues of the primary flagellar (*flag*-1) locus proteins, lacking the chemotaxis genes *cheZYBR-cheAW*, as well as the flagellar biosynthetic and regulatory genes *flhE, flgM* and *fliATZ* (Fig. 6). As such, the resultant flagellar system would be expected to be non-functional as is the case in the endosymbiotic *Sodalis* spp. However, gene expression analysis of the *fliC* (flagellin) and *motA* (motor protein A) genes and immunohistochemistry analysis with flagellins-specific antibodies showed the expression of both and production of flagellin in intrauterine larvae and the milk gland cells of tsetse flies, suggesting an important role for the *flag*-4 flagellum in *Wigglesworthia* vertical transmission from host mother to progeny [25]. Similarly, *Biostraticola tofi* DSM 19580, isolated from biofilm on a tufa limestone deposit has been shown to synthesise flagella and be capable of swimming motility [26]. As the genome of this bacterium solely incorporates a *flag*-4 locus, a role for the flagellar system it encodes in motility can be suggested for the *Biostraticola* and *Wigglesworthia.*

### The *flag*-5 locus is unique to *Plesiomonas shigelloides* among the Enterobacterales and codes for a polar flagellum

**Fig. 7:**
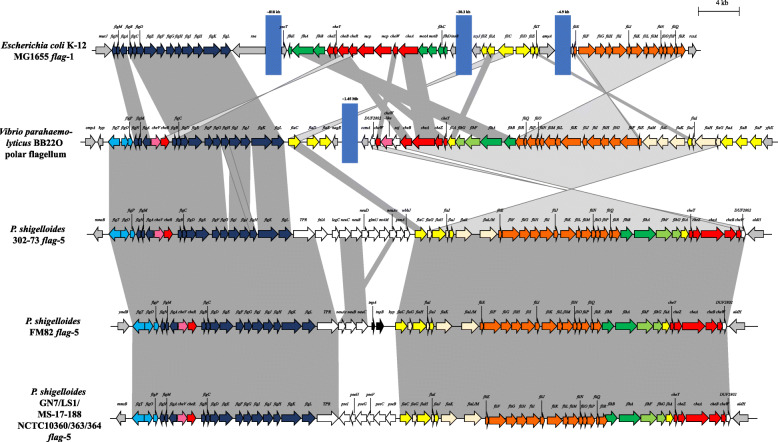
Schematic comparison of the *flag*-5 loci and *V. parahaemolyticus* BB22O polar flagellar locus. The *flag* loci genes are coloured according to orthology to conserved genes in the *flag*-1 locus, with flanking genes coloured in grey, non-conserved cargo genes in white and transposase genes in black. The blue blocks indicate chromosomal gaps between the *flag*-1 locus regions. The scale bar (4 kilobases) indicates the relative sizes of the loci

*Plesiomonas shigelloides* lack *flag*-1 loci, but previous studies have identified a lateral *flag*-2 locus in this species [15, 16]. Furthermore, they incorporate the distinct *flag*-5 locus that forms a separate clade in the SCO phylogeny (Fig. 1). The proteins encoded by this locus share limited sequence identity with those encoded on the other *flag* loci, with AAI values ranging between 32.80% (*flag*-4) and 34.92% (*flag*-1) across the 25 SCOs conserved among the loci (Additional file 1: Table S2). Instead, they share 54.95% AAI across 52 proteins with the polar flagellar loci of *Vibrio parahaemolyticus* BB22O (AF069392.3 and U12817.2) [16]. Furthermore, the *flag*-5 locus shows extensive synteny with the polar flagellar locus of the latter strain (Fig. 7). While the *V. parahaemolyticus* polar flagellar system is encoded by two loci, which are separated by ~ 1.45 megabases on the chromosome, the genes for polar flagellar synthesis in *P. shigelloides* are harboured in a single locus, which ranges between 57.9 and 62.0 kb in size and codes for 57–61 distinct proteins among the eight *P. shigelloides* incorporated in this study. These loci also represent the largest among the *flag*-1 to *flag*-5 loci.

*Vibrio parahaemolyticus* encodes two flagellar systems which facilitate movement under distinct conditions, with the lateral flagella (multiple) allowing swarming motility across surfaces, while the single polar flagellum enables swimming in liquid environments [7, 27]. The former are powered by the proton motive force, as observed for the peritrichous (*flag*-1) flagella of the Enterobacterales, while the latter derive their energy through the sodium membrane potential [7, 27]. A previous study has shown that *P. shigelloides* is likewise capable of both swarming and swimming motility, with the *flag*-2 and *flag*-5 linked to the former and latter form of motility, respectively [16].

Comparison of the protein complement with those of the other Enterobacterales *flag* loci identified twenty-nine proteins that are unique to the *flag*-5 loci, although seven of these share orthology with proteins encoded by the *V. parahaemolyticus* locus. Three of these orthologues, FlgO, FlgP and FlgT (30.04–48.83% AAI with *V. parahaemolyticus* BB22O AGB09241.1–244.1) are predicted to form part of the H ring, an additional basal body ring that is associated with the outer membrane. The H-ring is specific to *Vibrio* spp. and facilitates outer membrane penetration and external assembly of the sheathed polar flagella [27, 28]. The *Vibrio* polar flagellar locus lacks the genes *flhC* and *flhD* coding for the master transcriptional regulators in the enterobacterial peritrichous flagella. Instead the *Vibrio* polar flagellar loci incorporate three genes, *flaK, flaL* and *flaM* which are purported to fulfil this function. FlaK is a σ^54^-dependent transcription factor of FlaL and FlaM. The histidine kinase-like FlaL protein then phosphorylates FlaM which activates the transcription of the flagellar middle class genes [10]. The *Plesiomonas* FlaK orthologues share 58.15% AAI with that of *V. parahaemolyticus* BB22O (AGB10537.1). However, instead of two distinct FlaL and FlaM proteins, the *flag*-5 locus of *P. shigelloides* encodes a single 558 aa protein, FlaLM, with a histidine kinase domain (cd00082; Bitscore: 41; E-value 8.47e-5) and a σ^54^-activator domain (pfam00158; Bitscore: 295; E-value: 3e-98) at the C and N terminal, respectively. The FlaLM protein appears to be the product of a gene fusion between *flaL* and *flaM*, with the first 120 aa sharing 56.08% AAI with the *V. parahaemolyticus* FlaL protein (AGB10535.1; aa 1–184) and aa 200–558 sharing 59.24% AAI with the *V. parahaemolyticus* FlaM protein (AGB10536.1; aa 121–468). Both the *P. shigelloides flag*-5 and *V. parahaemolyticus* BB22O polar flagellar loci lack orthologues of the chaperone protein FliT. However, both incorporate a gene, *flaI*, adjacent to the *fliS* orthologues, which encodes a protein that is similar in size to FliT and hence may perform the chaperone function [7]. Furthermore, both encode a chemotaxis related protein, CheV, a CheY-CheW hybrid protein which is absent in the Enterobacterales *flag* loci [7].

A key difference between the *P. shigelloides flag*-5 loci and the *V. parahaemolyticus* BB22O polar flagellar locus is the presence of six distinct orthologues of the flagellin protein, FlaA-F in the latter strain. The *P. shigelloides flag*-5 loci include one orthologue, which shows highest sequence identity with the FlaC protein in *V. parahaemolyticus* BB22O (AGB09262.1; 51.85% AAI). By contrast, the *P. shigelloides flag*-5 loci uniquely incorporate between four and eleven genes adjacent to the *flaC* gene (Fig. 7; Additional file 1: Table S5). In *P. shigelloides* 302–73, these genes have been shown to code for proteins involved in the synthesis of the legionaminic acid, which posttranslationally glycosylates the flagellin protein [16]. Mutagenesis shows this glycan to be essential for biosynthesis of the flagellum in this strain [16]. Distinct flagellin glycosylation proteins are encoded in the other flag-5 loci. *P. shigelloides* FM82, incorporates three genes coding for the proteins NeuAc (acylneuraminate cytidylyltransferase), NeuB (N-acetylneuraminate synthase) and NeuC (UDP-N-acetyl-D-glucosamine 2-epimerase), involved in the synthesis of neuraminic acid, the precursor for legionaminic acid (Additional file 1: Table S5). These proteins share only 31.16% AAI with its orthologues in the *P. shigelloides* 302–73 *flag*-5 locus, suggesting that a distinct flagellin glycan is present in *P. shigelloides* FM82. The six other strains incorporated in this study code for orthologues of PseB (UDP-N-acetylglucosamine 4,6-dehydratase), PseC (UDP-4-amino-4,6-dideoxy-N-acetyl-beta-L-altrosamine transaminase), PseF (pseudaminic acid cytidylyltransferase), PseG (UDP-2,4-diacetamido-2,4,6-trideoxy-beta-L-altropyranose hydrolase), PseH (UDP-4-amino-4,6-dideoxy-N-acetyl-beta-L-altrosamine N-acetyltransferase) and PseI (UDP-4-amino-4,6-dideoxy-N-acetyl-beta-L-altrosamine N-acetyltransferase) (Additional file 1: Table S5), the six enzymes which constitute the pathway for the synthesis of pseudaminic acid that forms part of the flagellin glycan in a number of both Gram-negative (*Campylobacter* and *Helicobacter* spp.) and Gram-positive (*Geobacillus* spp.) taxa [29, 30]. These proteins share 97.41% AAI among the six *P. shigelloides flag*-5 loci that code for them, with the exception of PseI, where the orthologue in *P. shigelloides* NCTC 10360 shares only 29% AAI with those in the other five strains (97.42% AAI). This highlights that, while flagellin glycosylation appears to be a universal feature of the *P. shigelloides flag*-5 system, it is highly versatile both in the type of sugar and decoration of the glycan.

## Discussion

Flagellar motility, a common feature among most members of the Enterobacterales, was long considered to derive from a single chromosomally encoded peritrichous flagellar system (*flag*-1). However, a second, distinct lateral flagellar system (*flag*-2) was recently identified and shown to be fairly common across most family lineages within the order [14, 15]. Here we have identified three additional distinct flagellar loci, *flag*-3 to *flag*-5, with discrete taxonomic distributions. These loci range in size between 31.6 and 62.0 kb and code for between 36 and 61 proteins. Research on the *flag*-1 system in *E. coli* showed that the assembly of the thirty proteins (including ~ 20,000 copies of the filament protein FliC) that make up the primary flagellum structure is an energetically expensive process, contributing ~ 2% of the total energy burden of the cell [3]. Furthermore, rotation of the flagellum consumes ion motive forces which could power other cellular processes [13, 31]. As such, flagellar synthesis and motility is a tightly regulated at multiple levels [13, 31]. Furthermore, proteins involved in flagellar biosynthesis and function contribute on average 1.08% to the overall proteome (number of distinct flagellar proteins/total number of distinct proteins encoded on genome) and this already substantial proportion increases on average to 2.06% and up to 3.20% of the proteome when two or three *flag* loci are present, respectively. Thus the maintenance and functioning of two and sometimes three distinct flagellar systems would substantively increase the metabolic burden on the cell and raises questions on their evolution, functioning and biological roles among members of the Enterobacterales.

Previous studies have postulated that the primary (*flag*-1) flagellar system evolved from a single precursor or a few genes, which have subsequently undergone gene duplications and gene fusions to give rise to the current complement of genes required for flagellar synthesis. This is based on the extensive sequence conservation observed among twenty-four core *flag*-1 proteins in 41 motile taxa across eleven bacterial phyla [6, 32]. Similarly, the minimal gene set purported to have formed the primary flagellar system may have been duplicated elsewhere in the genome of many of the Enterobacterales. This may have given rise to the distinct clades represented by 1) the *flag*-1, − 3 and − 4 loci, 2) the *flag*-2 loci and 3) the *flag*-5 loci, which may subsequently have diversified through the recruitment of novel genes. For example, the *flag*-2 loci uniquely incorporate genes coding for the regulatory proteins LafK and LafZ and the hook-associated protein LafW, while the *flgO, flgP* and *flgT* genes coding for the H-ring and the regulatory genes *flaK* and *flaLM* are unique to *flag*-5 loci. This evolutionary hypothesis seems plausible for the *flag*-2 locus, which was previously shown to be prevalent among most enterobacterial lineages prior to extensive loss through deletion events [15]. By contrast, the polar *flag*-5 locus is unique among the Enterobacterales to strains of *P. shigelloides*, suggesting it may have been acquired through horizontal gene transfer, possibly from a member of the *Vibrionaceae*. The *flag*-1, − 3 and − 4 loci show a more convoluted relationship. These three loci also show extensive sequence conservation and synteny, with three gene blocks, *flgNMABCDEFGHIJKL, fliEFGHIJKLMNOPQR* and *fliCDST* (Fig. 3) universally present in each, while the *flag*-1 locus *flhEAB-cheZYBR-cheAW-motAB-flhDC* gene block is interspersed between these conserved blocks in the *flag*-3 and -4 loci. It is thus likely that the latter loci have been derived through duplications of the gene blocks and integration into an available genomic site, albeit in a different order from the *flag*-1 system. Notable is that the *flag*-4 loci in the three *Sodalis* spp. incorporated in this study display greater synteny with the *flag*-1 loci in these taxa, rather than the stereotypical *flag*-1 system as observed in *E. coli* K-12 str. MG1655 (Fig. 6). However, on the basis of sequence conservation, the *Sodalis flag*-1 locus is closer to its *flag*-1 counterpart, with the protein complement of the intact *S. praecaptivus* HS1 *flag*-1 locus sharing 59.2% AAI with the *E. coli* K-12 str. MG1655 *flag*-1 locus, while only sharing 47.5% AAI with the *S. praecaptivus flag*-4 locus. This suggests that, at least in *Sodalis*, the *flag*-1 was duplicated in its entirety, prior to sequence divergence of the *flag*-4 locus.

On the basis of the protein content encoded by the loci and their phylogenetic clustering, it was predicted that these additional flagellar loci code for two peritrichous (*flag*-3 and *flag*-4) and polar (*flag*-5) flagellar systems. A number of studies have shown the co-existence of two flagellar systems, particularly in *Vibrio* and *Aeromonas* spp., which host both a polar and lateral flagellar system that facilitate movement in liquid environments and across surfaces, respectively [2]. It can be envisaged that the primary (*flag*-1) peritrichous and lateral (*flag*-2) flagellar systems enable similar interchangeable modes of motility in the Enterobacterales. Similarly, the lateral (*flag*-2) and polar (*flag*-5) flagellar loci have been shown to facilitate swarming and swimming motility in *P. shigelloides*, respectively [16]. The combination of potentially dual peritrichous flagellar systems (*flag*-1 and *flag*-3; *flag*-1 and *flag*-4), however, is more puzzling. One possibility is that the second copy may serve as a source for spare-parts should the *flag*-1 locus no longer be functional. This is plausible among the *Sodalis* spp., where for example, the *flag*-1 of S*. glossinidius* ‘morsitans B4’ and *S. pierantonius* SOPE have undergone extensive gene decay, deletion and disruption through transposon integration (Fig. 6), although this degeneration is also evident in the *flag*-4 loci. However, the relatively low sequence conservation between the *flag*-1 and *flag*-3 (52.23% AAI) and the *flag*-1 and *flag*-4 (49.69% AAI) loci proteins suggest they may not be interchangeable. The main flagellar filament component, flagellin, is highly immunogenic and, upon recognition by host receptors, trigger both local and systemic innate and adaptive immune responses against bacteria in both plant and animal hosts [4]. Host-associated bacteria have evolved a number of means to counteract this process, including phase variable expression of flagellins with distinct antigenic properties and posttranslational glycosylation of the flagellin protein. Given that many of the enterobacterial taxa which are predicted to encode two peritrichous flagellar systems originated from animal and plant hosts, it is possible that the *flag*-1 and *flag*-3 loci are expressed under distinct conditions. It can also not be excluded that the *flag*-3 locus-encoded system plays a role other than motility. The *flag*-1 system of several pathogenic bacteria has, for example, been shown to serve as secretion system for several distinct virulence factors, while the presence of predicted secretion targets in the cargo regions of *flag*-2 loci may indicate a similar role for this lateral flagellar system [15, 33, 34]. Finally, previous analyses, of the *flag*-2 loci in particular, have highlighted the prevalence of transposable elements, pseudogenes and *en bloc* gene deletions within additional flagellar loci [14, 15]. It is possible that disruptions in the additional *flag* loci, may have resulted in non-functionality of previously functioning flagellar systems and concomitant energy conservation. It must be noted, however, that while transposase genes, pseudogenes and *en bloc* deletions are prominent features of the *flag*-4 loci, they are restricted to a very small number of *flag*-3a (11/155–7.1%) and *flag*-3b (6/95–6.3%) loci, while all *flag*-5 loci appear to be intact. Further characterization and knock-out mutagenesis of the additional flagellar systems need to be undertaken to unravel their functionality and biological roles. Furthermore, the cellular placement of the *flag*-3 and *flag*-4 loci was predicted purely on the basis of in silico data in this study, and further microscopic evaluation is required to determine their number and cellular localization of the flagella they encode.

## Conclusions

A comprehensive comparative genomic analysis showed that supernumerary flagellar systems represent a relatively common feature among members of the order Enterobacterales with one fifth of the enterobacterial taxa harbouring a lateral (*flag*-2), predicted peritrichous (*flag*-3 and *flag*-4) or polar (*flag*-5) locus on their genome, in addition to the primary (*flag*-1) flagellar system. Furthermore, a limited number of enterobacterial taxa incorporate loci coding for three distinct flagellar systems, which has to date not been reported for any bacterium. Considering the energetic burden of maintaining and operating multiple flagellar systems, it is possible that they play important biological roles in the Enterobacterales and may provide competitive advantage to the bacteria that possess them.

## Methods

### Identification of additional flagellar systems among the Enterobacterales

The presence of additional flagellar loci was determined on the same dataset of 4028 genomes representative of the order Enterobacterales as previously used to study the enterobacterial *flag*-2 loci [15]. Novel flagellar loci were identified by local tBlastN analyses with the *flag*-1 and *flag*-2 flagellar protein datasets from *E. coli* K-12 strain MG1655 (NCBI Acc. # U00096.3; 47 proteins; FlgNMABCDEFGHIKL-FlhEAB-CheZYBRWA-MotAB-FlhDC-FliZACDST-FliEFGHIJKLM NOPQR) and *E. coli* 042 (NCBI Acc. # CR753847.1; 38 proteins; LfhAB-LfiRQPNM-LafK-LfiEFGHIJ-LfgNMABDEFGHIJKL-LafWZABCDEFSTU) with BioEdit v.7.2.5 [35]. Genomic contigs where Blast hits were obtained were searched up- and down-stream to identify the full *flag* loci. The G + C% content of each of the loci was calculated using BioEdit v.7.2.5 [35]. Proteins encoded on the loci were predicted using the Prokaryotic GeneMark.hmm v.2 server [36] and functionally annotated using BlastP analyses against the National Center for Biotechnology Information (NCBI) non-redundant protein sequence database and the Conserved Domain Database using Batch CD-Search [37].

### Comparative and phylogenetic analyses

A representative subset of *flag*-1 and *flag*-2 loci, comprising three of each loci per genus in which they occur, was selected for comparative analyses. Orthologous proteins conserved among the representative *flag*-1 and *flag*-2 and the *flag*-3 to *flag*-5 loci were identified using Orthofinder 1.1.4 [38]. The 25 orthologous proteins occurring in single copy (SCOs) that were conserved among all the *flag* loci were utilized to calculate average amino acid identity (AAI) values between and within the different locus groups using compareM v 0.1.0 [39]. The SCOs were furthermore individually aligned using the M-Coffee implementation of T-Coffee [40]. The resultant proteins were concatenated and the alignment was curated using GBlocks v 0.91b [41]. This curated alignment was then used to construct a ML phylogeny with IQTree v 1.6.11 [42] with the appropriate evolutionary model predicted using Modelfinder [43] and phylogeny support through bootstrapping with UFBoot2 (*n* = 1, 000 replicates) [44]. Similarly, a ML tree was generated on the basis of 45 SCOs conserved among the *flag*-3a and *flag*-3b loci. Finally, a ML on the basis of four conserved house-keeping markers, GyrB, InfB, RecA and RpoB was generated as previously described [15] and used to map the prevalence of the novel *flag*-3, *flag*-4 and *flag*-5 loci.

## Supplementary information

**Additional file 1: Table S1.** Presence/absence of the *flag*-1 to *flag*-5 loci among the Enterobacterales. The presence of a specific locus is indicated by the contig/chromosomal sequence NCBI accession number. The previous nomenclature, isolation source and lifestyle/habitat of the 4028 studied enterobacterial taxa is indicated. **Table S2.** Average Amino Acid Identity (AAI) values (%) between the distinct *flag* loci. The AAI values were calculated on the basis of 25 SCOs conserved among all *flag* loci using CompareM [39]. A total of three representative taxa were selected for each genus for the *flag*-1, *flag*-2 and *flag*-3 (a and b) loci to avoid overrepresentation of taxa where the *flag* loci are particularly prevalent, while all *flag*-4 and *flag*-5 loci were included. **Table S3.** Molecular architectures of the *flag*-3, *flag*-4 and *flag*-5 loci among the Enterobacterales. The sizes, number of protein coding sequences (CDSs) encoded on the loci, G + C contents (%) and G + C deviation (%) from the average genomic G + C content are indicated. Where present, the sizes, locations and number of cargo proteins encoded on variable regions within the loci are shown. **Table S4.** Functional annotations of the cargo proteins encoded within variable regions in the *flag*-3 loci. The prevalence of each protein in the *flag-*3a and *flag-*3b loci are shown, as are the genera in which each protein is found. The AAI values (%) between the *flag*-3 orthologues, the average protein sizes, presence of conserved domains (with e-value and bitscore) and predicted functions are given. **Table S5.** Functional annotations of the cargo proteins encoded within variable regions in the *flag*-5 loci. The prevalence of each protein among the *flag*-5 loci of the distinct *Plesiomonas* spp. is indicated. The AAI values (%) between the *flag*-5 orthologues, the average protein sizes, presence of conserved domains (with e-value and bitscore), top non-*Plesiomonas* BlastP hits in the NCBI non-redundant protein database (with amino acid identity, e-value and bitscore) and predicted functions are shown.

## Data Availability

All data related to this publication are publicly available. The nucleotide and protein sequences from the reference strains for the *flag*-1 locus (*E. coli* K-12 strain MG1655), *flag*-2 locus (*E. coli* 042) and the two loci which comprise the polar flagellar system of *Vibrio parahaemolyticus* BB22O are available via the National Centre for Biotechnology Information (NCBI) nucleotide database (http://www.ncbi.nlm.nih.gov/nuccore/) under the accession numbers U00096.3, CR753847.1 and AF069392.3 and U12817.2, respectively. The NCBI nucleotide accession numbers for the contigs/chromosomes on which the target loci were found in the strains analysed in this study are indicated in Additional file 1: Table S1 and Table S3. Similarly, the top protein (BlastP) hits from Additional file 1: Table S5 are available via the NCBI protein database (http://www.ncbi.nlm.nih.gov/protein). The nucleotide as well as predicted protein sequences for the novel *flag*-3, *flag*-4 and *flag*-5 loci (as well as the flag-1 and *flag*-2 sequences used for comparative purposes) and the phylogenetic data (SCOs, aligned SCOs as well as raw phylogenetic trees) have been made publicly available via Researchgate (10.13140/RG.2.2.15622.60482).

## Abbreviations

AAI: Amino Acid Identity
CDS: Protein coding sequence
NCBI: National Centre For Biotechnology Information
ML: Maximum Likelihood
SCO: Single-Copy Orthologue
